# Research on the impact mechanism of environmental perception of stadium landscapes on sustainable spectatorship willingness from the perspective of embodied cognition: based on the experience model of “body–environment” interaction

**DOI:** 10.3389/fpsyg.2026.1827670

**Published:** 2026-05-20

**Authors:** Xiaoyu Ge, Nanjun Ouyang, Moyan Yang, Fengshan Yue, Wansu Chen, Shuqiao Meng, Wenxia Tong

**Affiliations:** 1 Northwestern Polytechnical University, Xi'an, China; 2 School of Art, Xi'an University of Architecture and Technology, Xi’an, China; 3 Department of Physical Education, Xidian University, Xi'an, China

**Keywords:** embodied cognition theory, environmental perception, spectator experience, stadium landscapes, willingness to continue watching games

## Abstract

**Introduction:**

The post-event utilization efficiency of stadiums and the sustained participation of spectators are core issues constraining the high-quality development of the competition and performance industry. In the era of the experience economy, spectators’ decision-making behavior is increasingly influenced by the interaction between environment and psychology. However, existing research has mostly focused on single dimensions such as service quality or customer satisfaction, lacking a theoretical framework that can systematically explain the complete path of “physical environment–psychological experience–behavioral intention”. From the perspective of embodied cognition, this study introduces the Stimulus-Organism-Response (S-O-R) theoretical framework, aiming to construct and test an integrated model to reveal the internal mechanism by which Environmental Perception of Stadium Landscapes affects Willingness to Continue Watching Games through multidimensional Spectator Experience.

**Methods:**

Based on questionnaire survey data from spectators of the Chinese Football Association Super League, this research empirically tests the impact of Environmental Perception of Stadium Landscapes and Spectator Experience on Willingness to Continue Watching Games using structural equation modeling and other methods.

**Results:**

The study finds that: (1) Environmental Perception of Stadium Landscapes has a significant positive direct impact on Willingness to Continue Watching Games; simultaneously, it also has a significant positive indirect impact on Willingness to Continue Watching Games through Spectator Experience. (2) Environmental Perception of Stadium Landscapes has a significant positive impact on Spectator Experience. (3) Spectator Experience has a significant positive impact on Willingness to Continue Watching Games. (4) In addition to direct effects, environmental perception indirectly impacts Willingness to Continue Watching Games through Spectator Experience.

**Discussion:**

This provides empirical evidence and theoretical reference for addressing the post-event operational challenges of stadiums in China in the future. It also points out the direction for the high-quality development of brand-oriented operations of stadiums in China in the future.

## 1 Introduction

Sports stadium, as the core spatial carriers for hosting large-scale events and mass sports activities, are undergoing a profound transformation in their value positioning (Cho et al., 2018). In the era of experience economy, audiences no longer go to Sports stadium just to watch the game itself, but to pursue a comprehensive sensory immersion and emotional experience (Shi, 2014). Therefore, how to maintain audience loyalty has become a hot research topic.

Previous studies have shown that the theories of service quality and customer satisfaction in marketing have been widely applied (Cronin et al., 2000), and tools such as SERVQUAL have been used to measure the dimensions of sports stadium services (Yoshida and James, 2010). In addition, Theory of Planned Behavior (TPB) has been used to predict attendance intentions, emphasizing subjective norms and perceived behavioral control (Jia et al., 2021). Although these mainstream frameworks have empirical utility, their research views the sports venue environment as a set of service attributes (such as seat comfort, bathroom accessibility) or aesthetic cues. They view the audience as passive information receivers or emotional responders, and the viewing experience is simplified as a psychological assessment of event content or service attributes (Hou et al., 2024). They assume that ‘better facilities’ linearly generate ‘better experiences’. However, this assumption cannot explain why a poorly functioning but culturally rich sports venue can outperform modern Sports stadium in cultivating audience loyalty. The research on this viewpoint also underestimates the influence path of the audience’s sustained willingness to participate through the interaction between the physical environment and the audience’s body, which generates contextualized experiences.

The theory of embodied cognition provides an alternative framework for explaining these anti empirical phenomena (Ceravolo, 2023). The embodied cognition theory that emerged from the second generation of cognitive science proposes that cognitive, emotional, and behavioral processes are rooted in the sensory motor system and arise from the dynamic coupling between the body and its physical environment (Cai et al., 2025). According to this viewpoint, watching a game live becomes an activity where the body is completely “present” and interacts in real-time with the venue space and atmosphere through multi-sensory collaboration (Jin et al., 2018). The spatial layout, visual landscape, acoustic envelope, and even the tactile sensation of the seats in Sports stadium are constantly in dialogue with the physical state of the audience, jointly shaping the transition from sensory perception to emotional fluctuations.

Due to the spatiality, physical presence, group atmosphere, and sensory immediacy of on-site sports viewing, it cannot be explained solely by “event quality, service evaluation, and viewing satisfaction”. The viewing experience is not an internal evaluation, but a real-time, multimodal emergent state generated through continuous physical environment interaction. Therefore, Sustainable Spectatorship Willingness is not only a reasonable plan, but also an action oriented tendency rooted in positive embodied memory. There is no evidence found in existing empirical research that examines sports venue landscape perception, viewing experience, and Sustainable Spectatorship Willingness as a complete mechanism. Based on the theoretical gaps and insufficient empirical evidence mentioned above, this study proposes from the perspective of embodied cognition: how can the physical and design attributes of sports stadium environments serve as interfaces for embodied interaction, shaping the audience’s physical perception and action possibilities, and systematically influencing their internal experiences and long-term behavioral tendencies? Exploring this issue not only deeply integrates environmental psychology, experiential research, and cognitive science, opening up a more explanatory theoretical frontier for sports audience research, but also provides highly actionable new principles for stadium design and management. In view of this, this study takes the Chinese Professional Football League as an example to construct and verify a comprehensive model based on embodied cognition theory, revealing the complete mechanism of “perception of sports stadium landscape environment - audience experience - willingness to continue watching the game”, thus laying a theoretical and empirical foundation for sports stadium operation and event experience design.

## 2 Literature review, theoretical analysis, and research hypotheses

### 2.1 Reconstruction of core concepts based on embodied cognition theory

Traditional research views spectator behavior as a disembodied process of information processing or emotional response, dissecting environment, experience, and willingness. This study introduces embodied cognition theory, advocating that cognition, emotion, and behavior arise from the real-time coupling and interaction between the body and the environment it is in. Based on this, we theoretically reconstruct the core concepts.

#### 2.1.1 Willingness to continue watching games: action orientation of embodied practice

The Willingness to Continue Watching Games is derived from attitude theory and behavioral prediction research (Moreno et al., 2016). It is typically defined as a predictive indicator of future behavior tendencies and is measured through multiple dimensions such as future attendance, recommendation, and loyalty (Biscaia et al., 2012). From an embodied perspective, this willingness is not only a rational evaluation of past experiences but also an action-oriented tendency rooted in bodily memories. When the audience’s body forms a positive perceptual-motor pattern and emotional state through successful interaction with a specific venue environment, this “embodied” memory generates an intuitive driving force to return to similar situations to reproduce or deepen the experience. Therefore, the Willingness to Continue Watching Games is essentially the body’s pursuit and commitment to meaningful interaction possibilities it can achieve.

#### 2.1.2 Spectator experience: dynamic emergence generated by the coupling of body and environment

Research on Spectator Experience often draws from the theories of experience economy and experience marketing (Brakus et al., 2009), and frequently references the multidimensional framework of brand experience, such as sensory, emotional, behavioral, and cognitive experiences described by Carlson et al. (2016). The embodied cognition theory provides a more fundamental philosophical foundation for this. This study argues that Spectator Experience is not the internalized reception of pre-existing event meanings, but rather the multimodal, integrated state generated in real-time through the continuous interaction of the spectator’s body with the specific physical-social environment of the venue (Hanh and Xuan, 2020). Sensory experience stems from the direct dialogue between bodily sensory channels and environmental stimuli; emotional experience is the physiological state of the body’s tendency to prepare for action in a given situation; behavioral experience involves the body’s exploration and participation in the environment; and cognitive experience is the narrative and meaning constructed based on the aforementioned embodied interactions. Together, they form an integral, emergent, and immersive whole.

#### 2.1.3 Environmental perception of stadium landscapes: physical assessment of “perception-action-possibility-stimulation”

Environmental perception is a crucial concept in environmental psychology, and research on consumer environments has also confirmed the influence of atmosphere on decision-making (Cronin et al., 2000). Within the embodied framework, environmental perception is redefined. The landscape and function of stadiums together form a network of “affordances” that is open to the audience’s bodies (Trail et al., 2003) = landscape elements (such as visual aesthetics and cultural symbols) primarily offer aesthetic and meaningful possibilities, inviting visual exploration and emotional engagement; functional elements (such as layout and facilities) mainly provide practical and actionable possibilities, supporting physical movement and comfortable participation. Therefore, environmental perception is a holistic and pre-reflective awareness and evaluation of the interactive possibilities that the environment offers to the audience’s body, related to the practice of spectating (Yoshida and James, 2010). It is not a passive reflection of objective attributes, but rather a preview of possibilities by the embodied subject, asking “What can I do and feel here?”

### 2.2 Theoretical analysis and hypothesis proposal

#### 2.2.1 Hypothesis on the impact of environmental perception of stadium landscapes on willingness to continue watching games

The perception of the stadiums environment may directly trigger behavioral intentions without the need for explicit experiential reflection. According to the perspective of “pre reflection” in ecological psychology and embodied cognition (Gallagher, 2005; Dreyfus, 2002), when facing an environment with high interactive potential, the body tends to have a tendency towards direct and pre reflection (Shenyang and Yutong, 2023). This tendency does not stem from the individual perception of sensory, emotional, or cognitive content, but rather from the environment as a whole directly “inviting” the body (Jia et al., 2021).

From a causal mechanism perspective, landscape attributes (such as iconic buildings, visual transparency, color and lighting atmosphere) mainly stimulate viewers’ longing through aesthetic attraction and spatial openness (Chen and Huang, 2025). Functional attributes such as seat comfort, accessibility, and facility convenience directly enhance the willingness to follow up by reducing physical activity costs and improving control. Both can be independent of the complete process of experiential reflection and directly have a positive causal effect on the willingness to continue watching the game. Therefore, we propose the first set of hypotheses.

*H1*: Stadium landscape perception (including landscape perception and functional perception) has a significant positive impact on the willingness to continue watching games.

Specifically, both landscape perception (H1a) and functional perception (H1b) are expected to positively influence continuance willingness.

#### 2.2.2 Hypothesis on the impact of environmental perception of stadium landscapes on the spectator experience

The material attributes of stadiums are not neutral backgrounds, but actively provide specific perceived and actionable resources (de Carvalho, 2020). A well-designed stadiums environment, through its landscape attributes such as visual signage, spatial layout, and ambient lighting, triggers targeted attention and emotional resonance among the audience, thereby directly shaping sensory and emotional experiences (Wong and Tang, 2016); By supporting the audience’s physical operations and task completion through stadiums functional attributes such as seat accessibility, flow fluency, and facility availability, it facilitates behavioral and cognitive experiences.

Specifically: landscape perception enhances the audience’s sensory experience by providing rich visual, auditory, and spatial stimuli; meanwhile, landscapes with aesthetic appeal or cultural symbolic significance can evoke positive emotions (Ge et al., 2021). In addition, landscapes may indirectly promote the audience’s body movements (such as cheering, waving) and cognitive construction of their sense of belonging by creating an overall participatory atmosphere.

Functional perception enhances the audience’s sense of fluency and control of behavior by reducing resistance to physical movement (such as convenient routes, comfortable seat temperature, clear signage) (Shi, 2014); smooth functional support can also reduce negative sensory interference and emotional fluctuations caused by inconvenience, thereby indirectly positively affecting sensory and emotional experiences. Meanwhile, reasonable functional design (such as information screens and interactive facilities) helps the audience form a clear understanding of the event process and stadiums rules.

In summary, we propose the second set of hypotheses and eight sub hypotheses.

*H2*: Stadium environmental perception positively affects spectator experience (including sensory, emotional, behavioral, and cognitive dimensions).

More detailed hypothesized paths are as follows.

Landscape perception → sensory, emotional, behavioral, cognitive experience (H2a–H2d).

Functional perception → sensory, emotional, behavioral, cognitive experience (H2e–H2h).

#### 2.2.3 Hypothesis on the impact of spectator experience on willingness to continue watching games

According to embodied cognition theory, when the audience experiences a positive and smooth viewing experience in a sports stadium, their body forms an implicit memory and preference that includes perception, emotion, behavior, and cognition (Martinette Kruger and Melville Saayman, 2012). This multidimensional experience will trigger the body’s desire and tendency to approach the same positive interactive state, directly driving the audience’s willingness to actively seek to repeat the experience in the future (Pine and Gilmore, 1998). The specific causal mechanism is as follows: (1) Sensory experiences (such as visual, auditory, and tactile richness and coordination) generate positive reinforcement through direct physical pleasure, making the audience willing to experience similar stimuli again. (2) Emotional experiences (such as excitement, sense of belon ṇging, and pride) establish emotional attachment through emotional memory, which is transformed into intrinsic motivation for repeated watching. (3) Behavioral experiences, such as cheering, jumping, and interacting with the surrounding audience, enhance self-efficacy through physical participation and encourage the audience to look forward to taking action again. (4) Cognitive experiences (such as understanding tactics, identifying with team culture, and gaining identity meaning) enhance personal value through meaning construction and drive long-term loyalty behavior. Therefore, we propose a third set of hypotheses.

*H3*: Spectator experience positively affects willingness to continue watching games.

Specifically, each of the four experience dimensions (sensory, emotional, behavioral, cognitive) is hypothesized to have a positive effect (H3a–H3d).

#### 2.2.4 Hypothesis on the mediating effect of spectator experience between environmental perception of stadium landscapes and willingness to continue watching games

The complete embodied cognitive pathway is a causal chain of “stimulus (environment)–experience (bodily sensation)–tendency (behavioral intention)”: environmental characteristics serve as antecedents, first triggering the Spectator Experience; And the Spectator Experience further serves as an intermediary mechanism, transmitting the influence of the environment to behavioral intentions (Meng and Alam, 2026). This means that the effect of stadiums environment on sustained viewing willingness is not entirely direct - some effects are indirectly achieved through the generation of Spectator Experience. In other words, Environmental Perception of Stadium Landscapes first triggers the interaction between the body and the environment (i.e., the Spectator Experience), which in turn drives the willingness to repeat behavior. If there is no “transmission” effect of the Spectator Experience, the direct effect of the environment on willingness may be weak or unstable. Therefore, the Spectator Experience plays a crucial mediating role in the “environment → intention” relationship. Based on this, we propose.

*H4*: Spectator experience mediates the relationship between stadium environmental perception and continuance willingness.

### 2.3 Theoretical model

**Figure 1:**
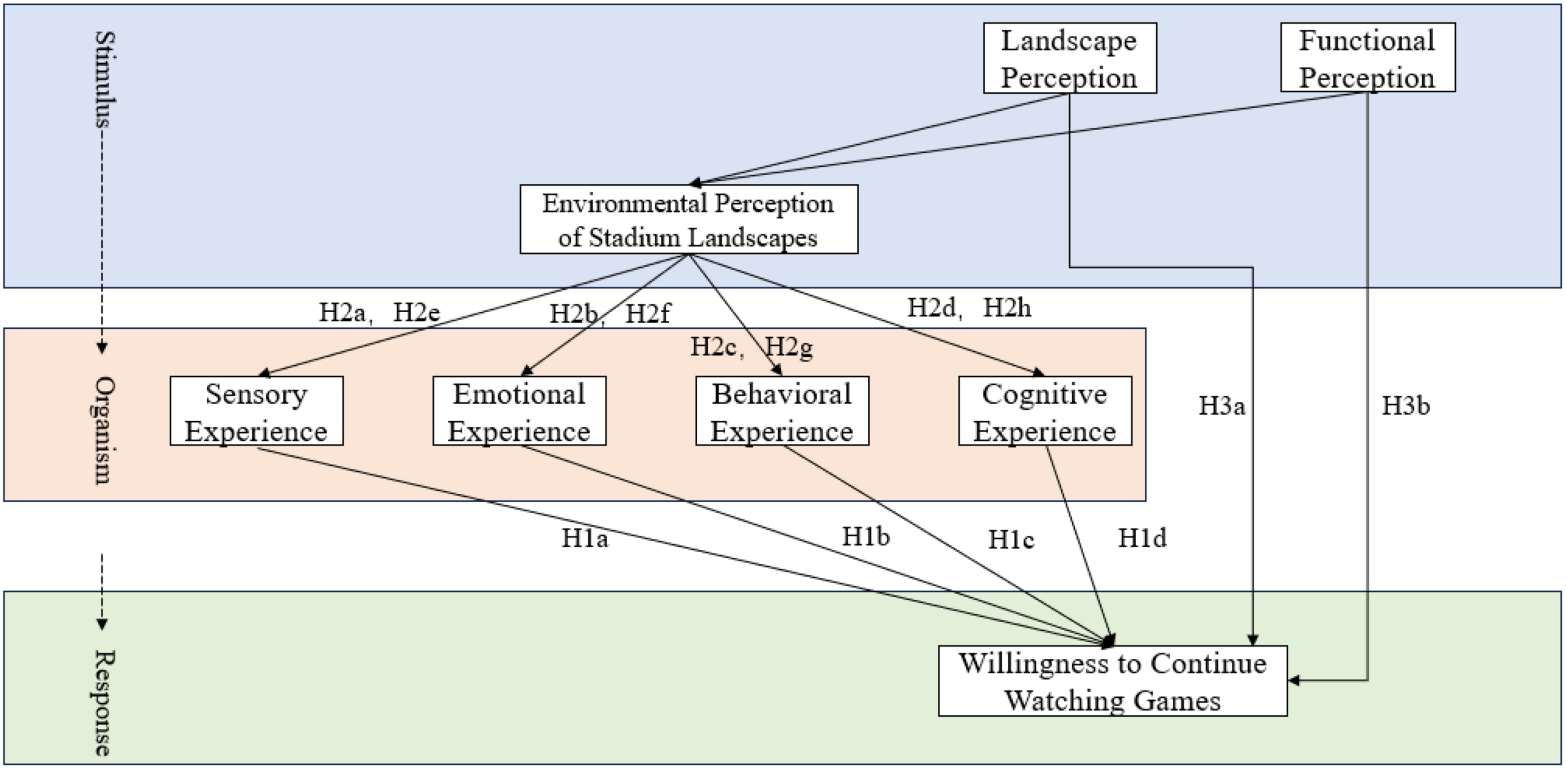
Theoretical model diagram.

In summary, the theoretical model constructed in this study is illustrated in Figure 1. This model depicts the embodied logical chain of “environment provides stimuli (S) → body generates experiences (O) → forms action tendencies (R)”, while retaining the potential direct effect path of the environment on willingness. This model aims to transcend the traditional “stimulus–response” or “cognitive evaluation” model, and understand spectator behavior within the dynamic generative process of “body embedded in context”. As shown in Figure 1.

## 3 Research design

### 3.1 Instrumentation

This study designed measurement items for variables by drawing on established domestic and international scales, complemented by expert interviews and pre-survey pilot testing. The resulting questionnaire includes four sections: stadium landscape environment, Spectator Experience, satisfaction, and Willingness to Continue Watching Games (Table 1). Specifically: Stadium landscape environmental perception adapts the scale developed by Liu et al. (2022), consisting of six items across two dimensions: landscape perception and functional perception. Spectator Experience uses Brakus et al.’s (2009) brand experience scale, with 12 items in four dimensions: sensory, emotional, behavioral, and cognitive experiences (3 items per dimension). Willingness to Continue Watching Games references Yoshida and James’ (2010) study, including three items measuring future attendance, event recommendation, and league loyalty (Yoshida and James, 2010). All items use a 5-point Likert scale ranging from “strongly disagree” (1) to “strongly agree” (5).

**Table 1:** Dimensions, item counts, and sources of measurement scales.

Scale	Dimensions	Items number	Examples of representative items	Reference sources
Environmental Perception of Stadium Landscapes	Landscape perception	6	Stadium provide rich atmosphere rendering with rich lighting landscapes	Liu et al. (2022)
	Functional perception		Stadium provides spacious and comfortable seats	
Spectator Experience	Sensory experience	12	This sports competition left a deep impression on my visual or other senses.	Brakus et al. (2009); Qi et al. (2015); Rongrong et al. (2018)
	Emotional experience		This sports competition can evoke my emotions and feelings.	
	Behavioral experience		I will participate in physical movements and behaviors while watching this sports competition	
	Cognitive experience		This sports competition has taught me new knowledge	
Willingness to Continue Watching Games	Future attendance, Recommending events, Remaining loyal to the event	3	I plan to continue watching Chinese Football Association Super League matches in the future	Yoshida and James (2010)

The original scales for spectator experience (Brakus et al., 2009) and willingness to continue watching games (Yoshida and James, 2010) were developed in English. Following the standard translation-back-translation procedure, two bilingual researchers independently translated the items into Chinese; a third researcher reconciled discrepancies. The Chinese version was then back-translated into English by a native English speaker and compared with the original. Minor wording adjustments were made to ensure semantic equivalence. For the stadium landscape environmental perception scale (Liu et al., 2022), which was originally published in Chinese, no translation was needed, but we culturally adapted several expressions for the Chinese Football Association Super League (CSL) context (e.g., “stadium signage” was specified as “club logo and sponsor banners”). To further enhance contextual relevance, we conducted cognitive interviews with 10 CSL spectators (5 season ticket holders, 5 occasional attendees). Based on their feedback, we reworded items that were ambiguous or not specific to live football settings.

### 3.2 Participants and procedures

*Sample selection*: Given the spatial scope of stadium landscapes defined in this study, participants were required to have attended at least one live sports event. To control for biases related to sport type and competition level, the study focused on spectators of the same-tier matches within the Chinese Football Association Super League (CSL). Additionally, to differentiate between “viewing intention” (attendance ≥ 0), “re-viewing intention” (attendance ≥ 1), and “sustained viewing intention” (attendance ≥ 2), the CSL was chosen due to its high match frequency and large spectator base.

In this study a multi-stage random sampling procedure was employed to ensure representativeness and randomness. First we uses a random number generator to randomly select a sample of matches from 31 to 14 rounds of competition. Secondly, on each selected competition day, the audience seating areas (such as east, west, south, and north stands) will be stratified, and a systematic random sampling method will be applied in each area. Thirdly, if the selected audience refuses or does not meet the qualifications, immediately replace the next seat and record this replacement. During the sampling process, there were no quota controls for gender, age, or education level.

*Questionnaire design screening*: The question “Have you attended at least 2 or more live sports events within the past year?” was set as a screening tool for survey respondents. To avoid potential homogeneity bias that may arise from the same person answering a set of questions, the questionnaire design included some reverse-phrased items and anonymous responses to reduce homogeneity bias in the valid data. To prevent the impact of unreasonable questionnaire design on the reliability and validity of the valid data, the questionnaire design and data collection were carried out in two steps, utilizing a cross-validation procedure for cross-sample testing of the structural model. During the pre-distribution process, all questionnaires were filled out face-to-face (between the interviewer and the respondent). On the basis of ensuring that each respondent was a fan of the Chinese Super League, 100 questionnaires were distributed on-site, and 82 valid questionnaires were collected, with an effective rate of 82%. At this stage, we optimized the wording of the items in the original questionnaire to address issues such as unclear semantics or difficulty in comprehension. Through item analysis and exploratory factor analysis, we reduced some items to form a formal questionnaire.

*Formal questionnaire structure*: Part 1: Demographic data (gender, age, education, occupation, income). Part 2: Scales for stadium landscape perception, Spectator Experience, and Willingness to Continue Watching Games. This study adopted a combination of purposive sampling and random sampling to distribute online questionnaires to fan groups of football clubs such as Wuhan Sanzhen, Shandong Mount Taishan, Zhejiang Professional, Beijing Guoan, and Shanghai SIPG from August to December 2025. Due to the relatively small number of live spectators in the first 30 rounds of matches, the questionnaire data mainly comes from 36 matches in the 31st to 34th rounds of the 2025 season. A total of 2000 questionnaires were distributed, and 1,362 were collected. After excluding those with inconsistent responses or identical choices, 1,284 valid questionnaires were obtained, with an effective response rate of 64.2%. The *t*-test indicated that there was no statistically significant difference between the invalid and valid questionnaires, suggesting that the non-response bias in the survey can be neglected. Data processing and analysis were primarily conducted using SPSS 24.0 and Amos 21.0 software. An analysis of the demographic characteristics of the valid samples (as detailed in the Table 2) revealed that the majority were males, aged 25 to 34, and had a bachelor’s degree. Additionally, this study conducted a Harman’s single-factor test on the formal questionnaire. Through exploratory factor analysis of the entire scale, it was found that the first factor explained 37.411% (less than 40%) of the variance without rotation, suggesting that most of the variance explained by a single factor does not exist, and the homogeneity bias is not severe.

**Table 2:** Basic sample demographics (*N* = 1,284).

Demographic variables	Category	Number	Effective percentage
Gender	Male	789	61.4%
	Female	495	38.6%
Age	20 years old (or younger)	142	11.1%
	2 L–30 years old	366	28.5%
	3 L–40 years old	429	33.4%
	4 L–50 years old	282	22.0%
	5 L–60 years old	54	4.2%
	Over 60 years old	10	0.8%
Education level	Below elementary school	0	0%
	Graduation from middle school	52	4.0%
	Graduated from junior college	247	19.3%
	Graduate with a bachelor’s degree	780	60.7%
	Graduate with a master’s degree	205	16.0%

### 3.3 Statistical analyses

The following statistical analysis methods were employed in this research: Descriptive statistical analysis: This method was used to analyze the basic characteristics of the sample data and the respondents who participated in the interviews and questionnaires. It covered demographic variables such as gender, age, and education level. Correlation analysis: To understand the strength of the association between the research variables, the Pearson product–moment correlation coefficient matrix was utilized to test whether there was a correlation between each pair of variables, serving as a test of the relationships among them. Exploratory Factor Analysis (EFA): It is a statistical technique that uses a small number of factors to explain a set of interrelated variables. In this study, the Principal Component Analysis(PCA) was adopted as the factor selection method. The Varimax rotation was applied to conduct exploratory factor analysis on the Environmental Perception of Stadium Landscapes, Spectator Experience, and Willingness to Continue Watching Games. Finally, this study used structural equation modeling to analyze the impact of environmental perception and audience experience of sports stadium landscapes on the willingness to continue watching the game. In the specific testing process, the measurement of variables is carried out using graded data as input indicators for latent variables, and the maximum likelihood method is used for structural equation model estimation.

## 4 Research results

### 4.1 Descriptive statistical results

The descriptive statistical analysis of the basic sample information is presented in Table 2. Regarding gender distribution, males accounted for 61.4%, while females made up 38.6%, indicating a relatively higher proportion of males. In terms of age, 33.4% of the respondents fell into the 31–40 age group, which was the largest segment, followed by the 21–30 age group with a proportion of 28.5%. With respect to education level, the majority of the respondents, 60.7%, had a bachelor’s degree, and 19.3% had an associate degree.

### 4.2 Reliability and validity test results

This study used the internal consistency method to assess the reliability of the collected sample data using Cronbach’s *α* coefficients. The specific analysis results are as follows Table 3.

**Table 3:** Reliability test results of sample data.

Variable	Number of items	Cronbach’s alpha
Landscape Perception	3	0.805
Functional Perception	3	0.891
Sensory Experience	3	0.854
Emotional Experience	3	0.832
Behavioral Experience	3	0.896
Cognitive Experience	3	0.873
Willingness to Continue Watching Games	3	0.801

From the test results in the Table 3, the *α* coefficients for the seven variables—landscape perception, functional perception, sensory experience, emotional experience, behavioral experience, cognitive experience, and Willingness to Continue Watching Games—all exceed 0.8. This indicates that the sample data collected from the 21 items of these seven variables meet the reliability assessment criteria, demonstrating high reliability and suitability for further analysis using structural equation modeling (SEM).

Next, this study analyzed the data from the 17 items in the formal questionnaire using the Kaiser–Meyer–Olkin (KMO) measure and Bartlett’s test of sphericity. The results are presented in Table 4.

**Table 4:** Kaiser–Meyer–Olkin (KMO) measure results and Bartlett’s test of sphericity results.

Kaiser-Meyer-Olkin (KMO) sample measure		0.875
Bartlett sphere test	Appro. chi-square	1229.874
	df	319
	Sig.	0.000

From the test results in Table 4, the KMO value of the sample data collected from the formal questionnaire was 0.875 > 0.5, indicating that factor analysis is appropriate. Bartlett’s test of sphericity showed a significant chi-square statistic with a significance level of 0.000 < 0.01, confirming the suitability for further factor analysis.

### 4.3 Factor analysis results

Based on the reliability and validity test results, exploratory factor analysis (EFA) was conducted to determine the factor structure of the variables. To examine the internal consistency of each factor variable, all observed variables were subjected to EFA. Factors were extracted using principal component analysis, with eigenvalues >1 as the inclusion criterion. Varimax rotation was applied, and factor loadings were retained if their absolute values exceeded 0.5. Factors were named according to their interpretability and loading magnitudes. The results of the exploratory factor analysis are presented in Tables 5–7.

**Table 5:** Exploratory factor analysis of environmental perception.

Scale Item	Factor load	Eigen value	Explanatory variance %	Accumulated explanatory variance %	Cronbach’s *α*
Landscape Perception		2.015	42.06	42.06	0.886
LP1	0.898				
LP2	0.824				
LP3	0.821				
Functional perception		1.436	26.97	69.03	0.875
FP1	0.844				
FP2	0.802				
FP3	0.873				

**Table 6:** Results of exploratory factor analysis of Spectator Experience.

Scale Item	Factor load	Eigen value	Explanatory variance %	Accumulated explanatory variance %	Cronbach’s *α*
Sensory Experience		4.336	30.54	30.54	0.903
SE1	0.738				
SE2	0.732				
SE3	0.698				
Emotional Experiences		2.990	18.30	48.84	0.903
FE1	0.831				
FE2	0.800				
FE3	0.759				
Behavioral Experience		1.842	12.28	61.12	0.883
AE1	0.834				
AE2	0.800				
AE3	0.754				
Cognitive Experience		1.372	9.14	70.26	0.801
CE1	0.848				
CE2	0.830				
CE3	0.812				

**Table 7:** Results of exploratory factor analysis of continuous viewing intention.

Scale Item	Factor load	Eigen value	Explanatory variance %	Accumulate explanatory variance %	Cronbach’s *α*
Willingness to Continue Watching Games		2.979	74.47	74.47	0.885
CW1	0.882				
CW2	0.861				
CW3	0.826				

The results of the exploratory factor analysis showed that the cumulative variance explained for the environmental perception variables in the questionnaire was 69.03, 70.26% for Spectator Experience variables, and 74.47% for Willingness to Continue Watching Games variables. Additionally, Cronbach’s *α* values for all seven latent variables exceeded 0.8, indicating that the questionnaire met the measurement requirements for the three constructs and that the latent variable divisions demonstrated high reliability. The data are suitable for structural equation modeling (SEM) analysis.

### 4.4 Pearson correlation analysis and normality test

Structural equation modeling typically requires observed variables to follow a multivariate normal distribution. This study conducted skewness and kurtosis tests on the measurement items of various latent variables. According to Table 8, the absolute skewness values of all items are between 0.152 and 0.789, and the absolute kurtosis values are between 0.201 and 0.956, both of which are far below the recommended standards (skewness < 2, kurtosis < 7). It can be considered that the data approximately follows a normal distribution and is suitable for model estimation using maximum likelihood method.

**Table 8:** Normality test results for each observation item (*N* = 1,284).

Latent variable	Item	Skewness	Kurtosis
Landscape Perception	LP1	−0.423	0.356
	LP2	−0.352	0.289
	LP3	−0.489	0.412
Functional Perception	FP1	−0.312	0.267
	FP2	−0.398	0.334
	FP3	−0.445	0.401
Sensory Experience	SE1	−0.467	0.423
	SE2	−0.412	0.489
	SE3	−0.401	0.378
Emotional Experiences	FE1	−0.478	0.412
	FE2	−0.323	0.367
	FE3	−0.389	0.345
Behavioral Experience	AE1	−0.245	0.223
	AE2	−0.289	0.256
	AE3	−0.312	0.278
Cognitive Experience	CE1	−0.366	0.312
	CE2	−0.402	0.345
	CE3	−0.389	0.334
Willingness to Continue Watching Games	CW1	−0.478	0.689
	CW2	−0.445	0.656
	CW3	−0.412	0.623

To explore the degree of linear correlation between latent variables, this study conducted Pearson product difference correlation analysis on the composite mean of each variable. According to Table 9, there is a significant positive correlation (*p* < 0.01) between landscape perception, functional perception, sensory experience, emotional experience, behavioral experience, cognitive experience, and willingness to continue watching the competition. The correlation coefficient ranges from 0.312 to 0.684, indicating a moderate to strong positive correlation between variables, which is suitable for further structural equation modeling analysis.

**Table 9:** Mean, standard deviation, and Pearson correlation coefficient matrix of each variable (*N* = 1,284).

Variable	Mean	SD	1	2	3	4	5	6	7
Landscape Perception	3.82	0.78	1						
Functional Perception	3.65	0.85	0.512**	1					
Sensory Experience	3.91	0.76	0.601**	0.498**	1				
Emotional Experiences	3.78	0.82	0.528**	0.462**	0.612**	1			
Behavioral Experience	3.43	0.91	0.362**	0.312**	0.445**	0.398**	1		
Cognitive Experience	3.51	0.88	0.387**	0.341**	0.469**	0.424**	0.523**	1	
Willingness to Continue Watching Games	3.95	0.73	0.552**	0.461**	0.684**	0.613**	0.438**	0.421**	1

** indicates *p* < 0.01 (dual tailed).

### 4.5 Model estimation results

This study included seven latent variables: landscape perception, functional perception, sensory experience, emotional experience, behavioral experience, cognitive experience, and Willingness to Continue Watching Games. Among these, landscape perception and functional perception were exogenous latent variables, while sensory experience, emotional experience, behavioral experience, cognitive experience, and Willingness to Continue Watching Games were endogenous latent variables. Each latent variable was measured by three observed variables, totaling 21 observed variables.

**Figure 2:**
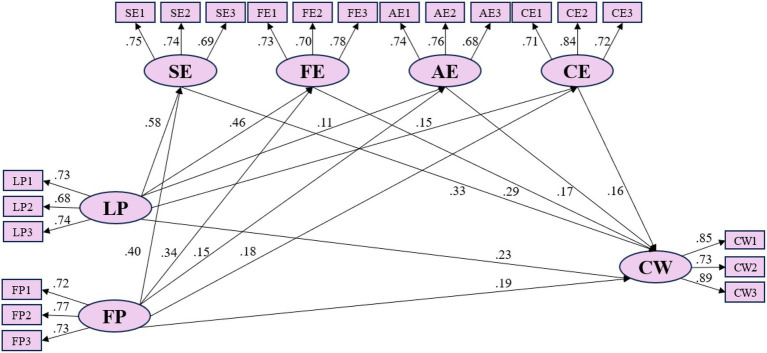
Structural equation model estimation results.

The SEM validated 15 hypotheses across four groups (H1–H4) to analyze the mechanisms through which stadium landscape environmental perception and Spectator Experience influence Willingness to Continue Watching Games. The SEM specification is visualized as follows Figure 2.

In the model specification, the two exogenous latent variables—landscape perception and functional perception—are mutually independent. Landscape perception has causal relationships with five endogenous latent variables: sensory experience, emotional experience, behavioral experience, cognitive experience, and Willingness to Continue Watching Games. Similarly, functional perception has causal relationships with the same five endogenous latent variables. The endogenous latent variables—sensory experience, emotional experience, behavioral experience, cognitive experience, and Willingness to Continue Watching Games—are mutually independent, with causal relationships existing between sensory/emotional/behavioral/cognitive experiences and Willingness to Continue Watching Games. Additionally, direct causal relationships are posited between landscape perception, functional perception, and Willingness to Continue Watching Games. Detailed parameter estimation results of the structural equation model are presented in Table 10.

**Table 10:** Test results of regression coefficients for each path in the model.

Path and hypothesis	Regression coefficient	Standardized regression coefficient	C.R.	*p*
Sensory Experience ← Landscape Perception	0.552	0.584	4.855	***
Emotional Experience ← Landscape Perception	0.426	0.461	6.370	***
Behavioral Experience ← Landscape Perception	0.102	0.110	2.921	***
Cognitive Experience ← Landscape Perception	0.150	0.152	3.449	***
Sensory Experience ← Functional Perception	0.389	0.403	1.388	***
Emotional Experience ← Functional Perception	0.331	0.343	2.244	***
Behavioral Experience ← Functional Perception	0.129	0.151	2.569	***
Cognitive Experience ← Functional Perception	0.143	0.182	3.476	***
Willingness to Continue Watching Games ← Sensory Experience	0.301	0.334	6.048	***
Willingness to Continue Watching Games ← Emotional Experience	0.256	0.293	6.230	**
Willingness to Continue Watching Games ← Behavioral Experience	0.133	0.172	3.274	**
Willingness to Continue Watching Games ← Cognitive Experience	0.138	0.161	2.445	***
Willingness to Continue Watching Games ← Landscape Perception	0.214	0.234	5.611	***
Willingness to Continue Watching Games ← Functional Perception	0.156	0.193	2.130	***
LP1 ← Landscape Perception	1.013	0.733		***
LP2 ← Landscape Perception	0.992	0.678	8.267	***
LP3 ← Landscape Perception	1.004	0.739	7.765	***
FP1 ← Functional Perception	0.982	0.721		***
FP2 ← Functional Perception	1.033	0.774	8.886	***
FP3 ← Functional Perception	0.989	0.728	9.604	***
SE1 ← Sensory Experience	1.012	0.748		***
SE2 ← Sensory Experience	1.014	0.743	7.724	***
SE3 ← Sensory Experience	0.987	0.692	7.246	***
FE1 ← Emotional Experience	0.974	0.732		***
FE2 ← Emotional Experience	0.985	0.699	7.948	***
FE3 ← Emotional Experience	1.044	0.784	7.667	***
AE1 ← Behavioral Experience	1.003	0.748		***
AE2 ← Behavioral Experience	1.051	0.767	10.041	***
AE3 ← Behavioral Experience	0.946	0.684	9.195	***
CE1 ← Cognitive Experience	0.962	0.713		***
CE2 ← Cognitive Experience	1.081	0.842	9.060	***
CE3 ← Cognitive Experience	0.966	0.719	9.407	***
WC1 ← Willingness to Continue Watching Games	0.847	0.851		***
WC2 ← Willingness to Continue Watching Games	0.731	0.733	11.807	***
WC3 ← Willingness to Continue Watching Games	0.887	0.890	10.198	***

*** indicates *p* < 0.001.

As shown in the Table 10, in the measurement model of this study, the *p*-values for all paths from latent variables (landscape perception, functional perception, sensory experience, emotional experience, behavioral experience, cognitive experience, Willingness to Continue Watching Games) to their observed variables were <0.01, passing significance tests. This indicates that the observed variables effectively measure the latent constructs.

For the structural paths: Sensory Experience ← Landscape Perception, Emotional Experience ← Landscape Perception, Behavioral Experience ← Landscape Perception, Cognitive Experience ← Landscape Perception, Sensory Experience ← Functional Perception, Emotional Experience ← Functional Perception, Behavioral Experience ← Functional Perception, Cognitive Experience ← Functional Perception, Willingness to Continue Watching Games ← Sensory Experience, Willingness to Continue Watching Games ← Emotional Experience, Willingness to Continue Watching Games ← Behavioral Experience, Willingness to Continue Watching Games ← Cognitive Experience, Willingness to Continue Watching Games ← Landscape Perception, Willingness to Continue Watching Games ← Functional Perception. All path coefficients had *p* < 0.01, confirming statistical significance at the *p* = 0.01 level. These 14 paths passed the significance tests. The specific initial model fit analysis results are as Table 11.

**Table 11:** Initial model fit analysis results.

Fit Index Type	Fit Index	Model value	Reference standard
Absolute Fit	*χ*^2^/df (chi-square/df)	1.626	1 < *χ*^2^/df < 2
	GFI (Goodness-of-Fit Index)	0.907	>0.90
	AGFI (adjusted GFI)	0.868	>0.80
	RMSEA (root mean square error of approximation)	0.057	<0.08
Relative Fit	CFI (Comparative Fit Index)	0.962	>0.90
	NFI (Normed Fit Index)	0.900	>0.90
	NNFI/TLI (Tucker-Lewis Index)	0.953	>0.90
	IFI (Incremental Fit Index)	0.963	>0.90

*Key findings:* Absolute fit indices: *χ*^2^/df = 1.626, GFI = 0.907, AGFI = 0.868, RMSEA = 0.057. Relative fit indices: *χ*^2^/df = 1.626, GFI = 0.907, AGFI = 0.868, RMSEA = 0.057. CFI = 0.962, NFI = 0.900, NNFI = 0.953, IFI = 0.963.

Evaluating the fitting degree of structural equation modeling (SEM) cannot be based solely on a single indicator, but requires following a rigorous set of logic. Its core idea is to make comprehensive judgments from multiple perspectives and criteria, from the overall model to local details, from absolute fitting to relative efficiency.

Firstly, based on the principle of integrating multiple criteria and types of indicators, this study examined two types of indicators: absolute fitting indicators and relative fitting indicators, totaling 8 indicators. The results show (Table 11) that these two indicators meet the standards, indicating that the overall fit of the model is within a reasonable range. Secondly, based on the principle of whole to part, after confirming that the overall model fit is acceptable, it is necessary to thoroughly examine the local details. This study examined the outliers of standardized coefficients and the critical ratio values for each path. The results show (Table 10) that the critical ratio of each path is within a reasonable range, and no outliers were found in the standardized coefficients. Therefore, it is considered that the local fitting degree of the model is within a reasonable range. Finally, based on the principles of sample sensitivity and model complexity, the standards can be appropriately relaxed. The large sample size of this study resulted in low sample sensitivity, thus reducing the value of the chi square index. In order to prevent overfitting caused by the complexity of the model, this study focuses on the approximate root mean square error index, while relaxing the GFI and AGFI indices.

On the basis of following the SEM evaluation logic and multiple evaluation principles, this study sorted out the model fitting analysis index table (Table 11).

The above indicator results show that the model fitting is reasonable, and the path coefficient results are reasonable and can reflect the influence relationship between variables.

### 4.6 Hypothesis testing results

Based on the proposed mechanism model of stadium landscape environmental perception and Spectator Experience on Willingness to Continue Watching Games, hypothesis testing was conducted. The criteria for significance were: Path coefficients with *p* < 0.05 (statistically significant). Path coefficients with *p* < 0.01 (highly significant). Results summary in Table 12.

**Table 12:** Hypothesis testing results.

Hypothesis	Path direction	Test results
H1a	Landscape perception has a significant positive impact on the willingness to continue watching matches	Valid
H1b	Functional perception has a significant positive impact on Willingness to Continue Watching Games	Valid
H2a	Landscape perception has a significant positive impact on sensory experience.	Valid
H2b	Landscape perception has a significant positive impact on emotional experience.	Valid
H2c	Landscape perception has a significant positive impact on behavioral experience.	Valid
H2d	Landscape perception has a significant positive impact on cognitive experience.	Valid
H2e	Functional perception has a significant positive impact on sensory experience.	Valid
H2f	Functional perception has a significant positive impact on emotional experience.	Valid
H2g	Functional perception has a significant positive impact on behavioral experience.	Valid
H2h	Functional perception has a significant positive impact on cognitive experience.	Valid
H3a	Sensory experience has a significant positive impact on the willingness to continue watching matches.	Valid
H3b	Emotional experience has a significant positive impact on the willingness to continue watching matches.	Valid
H3c	Behavioral experience has a significant positive impact on the willingness to continue watching matches.	Valid
H3d	Cognitive experience has a significant positive impact on the willingness to continue watching matches.	Valid
H4	The Spectator experience has a significant positive mediating effect between the perception of stadiums environment and the willingness to continue watching	Valid

## 5 Discussion of results

### 5.1 Analysis of environmental perception’s impact on willingness to continue watching games

The latent variables of landscape perception and functional perception have a significant positive impact on The Willingness to Continue Watching Games, with the impact coefficient of landscape perception being 0.23 and the impact coefficient of functional perception being 0.19. The research results indicate that improvements in environmental landscapes such as landscape design and stadium prop layout in stadium can effectively promote consumers’ willingness to continue watching sports events. Compared with functional renovations such as stand seats, bathroom decoration, and large screen decoration, renovations in the sports cultural landscape of the stadium, stadium slogans and logo landscapes, as well as event-related promotional props, can better promote consumers’ willingness to watch sports events (Hou et al., 2024). This empirical result forms a completely opposite conclusion compared to the leisure sports service industry (Shenyang and Yutong, 2023). This study believes that this is determined by the characteristics of sports event services. The core service product of large-scale sports events is the event viewing service; environmental landscape renovations such as stadium slogans and event promotional props surrounding the event viewing service have been deeply embedded into the quality of event viewing services (Quansah, 2022). Even if a game lacks these necessary environmental landscape facilities, even if the game is very exciting, fans still feel that the atmosphere of watching the game is lacking. Functional perception, on the other hand, is an accessory to the quality of event viewing services (Chen et al., 2025). For viewers, functional perception does affect their willingness to watch sports events, but good venue functional design is merely icing on the cake.

In addition to direct effects, environmental perception indirectly impacts Willingness to Continue Watching Games through Spectator Experience. Due to the complexity of indirect pathways, results are presented in Table 13.

**Table 13:** Summary of indirect effects.

Influence path	Indirect benefit	Total indirect benefit
Landscape Perception → Sensory Experience → Willingness to Continue Watching Games (0.58 × 0.33)	0.191	0.367
Landscape Perception → Emotional Experience → Willingness to Continue Watching Games (0.46 × 0.29)	0.133	
Landscape Perception → Behavioral Experience → Willingness to Continue Watching Games (0.11 × 0.17)	0.019	
Landscape Perception → Cognitive Experience → Willingness to Continue Watching Games (0.15 × 0.16)	0.024	
Functional Perception → Sensory Experience → Willingness to Continue Watching Games (0.40 × 0.33)	0.132	0.285
Functional Perception → Emotional Experience → Willingness to Continue Watching Games (0.34 × 0.29)	0.099	
Functional Perception → Behavioral Experience → Willingness to Continue Watching Games (0.15 × 0.17)	0.026	
Functional Perception → Cognitive Experience → Willingness to Continue Watching Games (0.18 × 0.16)	0.029	

Confidence interval of 95% and *p* < 0.05.

The results validate the research hypothesis of this study. This study posits that the environmental perception of stadium serves as a tool for event product providers to transform spectator services into something that customers can perceive and directly perceive the event brand through various means such as creating a venue’s environmental atmosphere, designing venue landscapes, and temporarily setting up and arranging stadiums (Schut and Glebova, 2022). This enables customers to better understand the connotations of the products and services provided by sports events (Galily et al., 2023). Consequently, it directly impacts customers’ Spectator Experiences and further influences their willingness to continue attending events. This provides empirical evidence and theoretical insights for the work of venue layout and setup in future venue operations and event hosting processes.

### 5.2 Analysis of environmental perception’s impact on spectator experience

The latent variable of landscape perception has a significant positive impact on the four latent variables of sensory experience, emotional experience, behavioral experience, and cognitive experience. The impact coefficients of landscape perception on the above four latent variables are 0.58, 0.46, 0.11, and 0.15, respectively. The research results indicate that the landscape environment of the venue can significantly affect consumers’ sensory experience and emotional experience. The sensory and emotional appeals of consumers during the game are consistent (Raymond et al., 2017). Landscape perception factors such as color and lighting in the stadium can affect sensory experience; environmental layout props such as cultural landscape settings and cultural propaganda slogans in the venue can affect emotional experience. However, landscape perception has only a weak impact on behavioral experience and cognitive experience. This study believes that this is due to the lack of distinctive features in venue construction. Taking the Chinese Super League as an example, fans travel to multiple cities to cheer for their teams, and the stadium layouts in different cities are uniform, making it difficult to form differences in fans’ cognition about the hosting of games in different stadiums (Shi, 2014). This also reflects, from one aspect, that the cultural personality and cultural sports tourism consumption vitality in various regions have not yet been activated in the operation process of stadium in China, and there is still considerable market development space.

Functional perception has a significant positive impact on the four latent variables of sensory experience, emotional experience, behavioral experience, and cognitive experience. The impact coefficients of functional perception on the above four latent variables are 0.40, 0.34, 0.15, and 0.18, respectively. The research results indicate that functional perception can significantly influence consumers’ sensory and emotional experiences, which is consistent with empirical research findings from current tourist attractions (Yoon et al., 2025). This suggests that essential functional and convenient facilities in stadiums can effectively enhance consumers’ sensory and emotional experiences, providing them with a sense of respect and comfort akin to being at home. However, functional perception only has a relatively weak impact on behavioral and cognitive experiences. This study believes that this is due to the lack of close integration between functional facilities and sports events. Currently, most stadium primarily prioritize safety in the design of barrier-free access, elevators, stands, restrooms, and food and beverage outlets, sacrificing numerous opportunities to incorporate event elements to meet safety standards. For example, food and beverage outlets mainly adhere to Western standards for processing cooked food and fast food, failing to provide products related to the event such as ice cream and local snacks characteristic of the event venue. This results in functional perception not deeply shaping the cognitive and behavioral experiences of spectators. In the future, sports venue operators can refer to the measures taken by subway station builders in combining station names with the renovation of facilities such as passageways, turnstiles, and elevators. They can develop event-related products centered around sports culture to enhance the innovation capability of venue brands.

### 5.3 Analysis of spectator experience’s impact on willingness to continue watching games

The four latent variables of sensory experience, emotional experience, behavioral experience, and cognitive experience have a significant positive impact on The Willingness to Continue Watching Games. The impact coefficients of the above four latent variables on The Willingness to Continue Watching Games are 0.33, 0.29, 0.17, and 0.16, respectively. The research results indicate that sports events are typical experience economy products, and around these events, services such as event viewing, broadcasting, cultural activities, and tourism have been formed. When consumers have a good sensory experience in the stadium, they are likely to develop a willingness to continue watching sports events (Eklund et al., 2024); as consumers’ involvement in sports events increases, especially after joining relevant sports fan groups, when they have a good emotional experience, it can also greatly influence their willingness to continue watching sports events (Ladouce et al., 2017). However, when consumers have behavioral and cognitive experiences, they can only influence their willingness to continue watching sports events to a smaller extent. This study believes that the reason lies in the deficiencies in the brand building of stadium and events, which are manifested in the failure to form deep-rooted brand recognition among consumer groups and the failure to create differentiated viewing or event cultures between different stadiums.

This result provides empirical evidence for the future strategy of high-quality operation of stadium. Currently, consumers in China generally exhibit a trend of rational consumption. Against the backdrop of changing consumption trends and the international trend of deglobalization, the sports venue operation industry in China faces dual challenges both domestically and internationally. Therefore, sports venue operators should pay more attention to service quality, Spectator Experience, and venue brand reputation. The original business model, which focuses on venue construction, functional development, and event introduction, should gradually shift to a high-quality business model centered on venue operation, venue culture shaping, and venue brand building. The development path of refinement and branding should gradually replace the original extensive path of scale economic development, thereby promoting the competition and performance industry to gradually enter a high-quality development stage oriented towards domestic consumption. It is worth noting that in this study the Willingness to Continue Watching Games variable was used to measure future intentions, while the Spectator Experience variable was measured retrospectively. This may lead to cross-sectional designs with different time reference points expanding the variance of common methods. In the future, this study will adopt a longitudinal study design to reduce measurement bias.

## 6 Research conclusion

Based on questionnaire data and using structural equation modeling, this study examined the impact of stadium landscape environmental perception and watching experience on the Willingness to Continue Watching Games. The results show: (1) Environmental perception has a significant positive direct impact on the Willingness to Continue Watching Games; additionally, environmental perception also exerts a significant positive indirect impact on this willingness through watching experience. (2) Environmental perception has a significant positive impact on watching experience. (3) Watching experience has a significant positive impact on the Willingness to Continue Watching Games. It should be emphasized that the sampling time of this study was during the recovery stage of the Chinese Super League viewing market, so it was found in the sample description that the characteristics of the sampled audience, such as age and education, were different from previous survey studies. But this also provides a reference for exploring the audience’s perception of the environment and willingness to watch games in different market stages. Future research will adopt a cross year longitudinal study design to further validate the conclusions of this study.

The main research conclusions are as follows: (1) This study constructs a theoretical model among environmental perception, watching experience, and Willingness to Continue Watching Games, providing empirical evidence and theoretical references for addressing future operational challenges of stadium in China. (2) The study identifies the mediating effect of watching experience between environmental perception and Willingness to Continue Watching Games; specifically, the mediating effects of sensory experience and emotional experience are stronger than those of behavioral experience and cognitive experience. These findings suggest that stadium operators may prioritize sensory and emotional enhancements, but future experimental research is needed to confirm causal priority.

While this study validates the theoretical model among environmental perception, watching experience, and Willingness to Continue Watching Games, it has certain limitations: (1) Although the conceptual model is validated, the unique complexity of fans’ continuous watching behavior warrants future inclusion of real-world behavioral data for revalidation. (2) The cross-sectional data analysis only reveals correlations between concepts; experimental methods should be used to further test the hypotheses. (3) The study focuses solely on the positive impact of watching experience on Chinese Super League fans’ willingness to continue watching, ignoring the inhibitory effects of negative experiences. Future research should incorporate negative experiences to enhance model completeness. (4) Due to funding constraints, the sampling scope was limited to spectators of the last four rounds of the 2025 Chinese Super League Expanding the project scope and sampling range is necessary.

This research adheres to a problem-oriented approach, combining theoretical guidance with empirical analysis of real-world issues. Based on these conclusions, studies examining the impact mechanisms of environmental perception, watching experience, and Willingness to Continue Watching Games from meso- and micro-perspectives can expand the application of theories related to venue experiences and spectator attitudes, promoting interdisciplinary research at the intersection of economics, management, and sports science. Additionally, these findings provide empirical support for resolving current operational challenges in Chinese stadiums and pave the way for high- quality development in stadium operations and the competition and performance industry.

## Data Availability

The raw data supporting the conclusions of this article will be made available by the authors, without undue reservation.
